# Dictyostelid Cellular Slime Molds from Christmas Island, Indian Ocean

**DOI:** 10.1128/mSphere.00133-19

**Published:** 2019-04-10

**Authors:** Pu Liu, Yue Zou, Wenxiu Li, Yu Li, Xinru Li, Songhao Che, Steven L. Stephenson

**Affiliations:** a Engineering Research Center of Chinese Ministry of Education for Edible and Medicinal Fungi, Jilin Agricultural University, Changchun, People’s Republic of China; b Department of Biological Sciences, University of Arkansas, Fayetteville, Arkansas, USA; Carnegie Mellon University

**Keywords:** Amoebozoa, *Cavenderia*, *Dictyostelium*, phylogeny, taxonomy

## Abstract

Christmas Island (10°30′S, 105°40′E) is an Australian external territory located in the Indian Ocean, approximately 350 km south of Java and Sumatra and about 1,550 km northwest of the closest point on the Australian mainland. In May 2017, 20 samples of soil/humus were collected on Christmas Island and processed for dictyostelid cellular slime molds. Four species were recovered. Two of these (Dictyostelium purpureum and Cavenderia aureostipes) are common and widely distributed throughout the world, but two other species (Dictyostelium insulinativitatis sp. nov. and Dictyostelium barbarae sp. nov.) were found to be new to science and are described here.

**IMPORTANCE** Reported here are the results of a study for dictyostelids carried out on Christmas Island, Indian Ocean. Six isolates representing four species of dictyostelid cellular slime molds were obtained from two of the four localities from which samples were collected on the island. Two of the species (*Dictyostelium insulinativitatis* and *D. barbarae*) belong to the Dictyosteliaceae, genus *Dictyostelium*, and are new to science. These are described based on both morphology and phylogeny. The diversity and abundance of dictyostelids on Christmas Island appear to be low, which might in part be due to the abundance of land crabs, which considerably reduce the extent of the litter layer on the forest floor.

## Abstract (precis)

Reported here are the results of a study for dictyostelids carried out on Christmas Island, Indian Ocean. Six isolates representing four species of dictyostelid cellular slime molds were obtained from two of the four localities from which samples were collected on the island. Two of the species (*Dictyostelium insulinativitatis* and *D. barbarae*) belong to the Dictyosteliaceae, genus *Dictyostelium*, and are new to science. These are described based on both morphology and phylogeny. The diversity and abundance of dictyostelids on Christmas Island appear to be low, which might in part be due to the abundance of land crabs, which considerably reduce the extent of the litter layer on the forest floor.

## INTRODUCTION

Dictyostelid cellular slime molds (dictyostelids) are single-celled, eukaryotic, phagotrophic bacterivores usually present and sometimes abundant in terrestrial ecosystems. These organisms represent a normal component of the microbiota in soils and apparently play a role in maintaining the natural balance that exists between bacteria and other microorganisms in the soil environment. The primary microhabitat for dictyostelids is the leaf litter decomposition zone of forest soils, but they also occur in other types of soils (1).

Dictyostelids have been reported from numerous localities throughout the world, but there are relatively few reports of the group from small isolated islands (2, 3). During the course of a survey for myxomycetes carried out on Christmas Island (S. L. Stephenson and B. C. Stephenson, submitted for publication) in May 2017, 20 samples of soil/humus for isolation of dictyostelids were collected by the senior author and sent to the first author for processing. Four species of dictyostelids were recovered from these samples, and two of these are species new to science. The primary objective of this paper is to describe these two new species.

**FIG 1:**
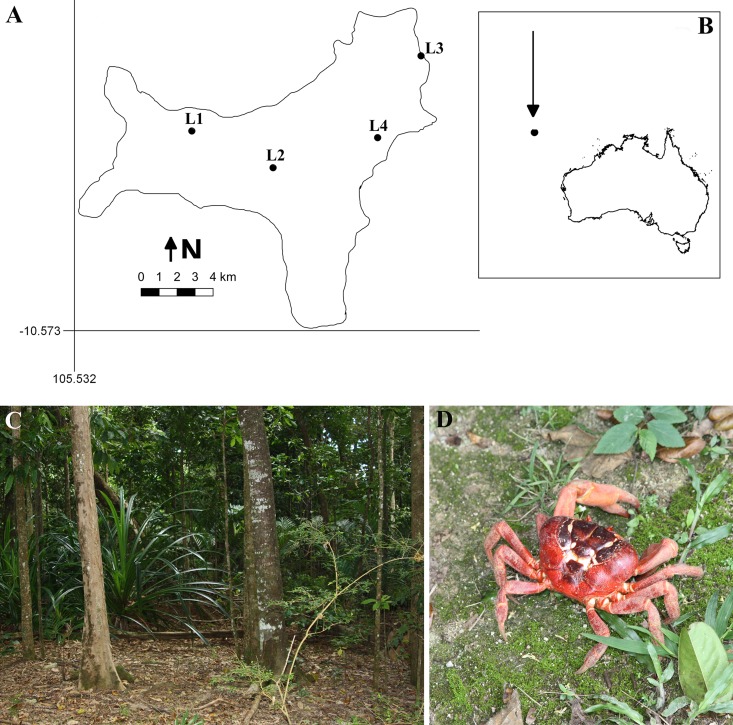
Christmas Island. (A) Map showing the collecting localities on the island. (B) Location of the island with respect to mainland Australia. (C) Typical tropical forest on the central plateau of the island. (D) Example of the large land crabs which are often exceedingly abundant in the forests on the island. (Maps courtesy of Carlos Rojas, reproduced with permission.)

Christmas Island (10°30′S, 105°40′E) (Fig. 1A) is an Australian external territory located in the Indian Ocean, approximately 350 km south of Java and Sumatra and about 1,550 km northwest of the closest point on the Australian mainland (Fig. 1B). It has a total area of 135 km^2^, and the highest point on the island is 361 m. Most of the island consists of a gently undulating plateau that represents the summit of an underwater mountain more than 4,500 m high. Christmas Island receives an average of 214 cm of rainfall each year, with the majority falling between November and June. Very little surface water exists on the island. Temperatures vary little throughout the year. The highest temperature is usually around 29°C in March and April, whereas the lowest temperature is about 23°C and occurs in August.

Much of the island is covered by tropical forests, with two types which are strongly correlated with soil depth. The predominant type is an evergreen tall forest that is found in areas characterized by deep soils. This includes most of the central plateau of the island (Fig. 1C). A semideciduous closed forest occurs in areas with shallow soils. A total of 213 species of native plants are known from Christmas Island, 17 of which are endemic to the island. However, approximately 250 species of exotic plants also have been introduced to the island (4). One of the more prominent of these is the coconut palm (Cocos nucifera L.).

The most conspicuous animals in the forests on Christmas Island are indigenous large land crabs (Gecarcoidea natalis Pocock), which are often exceedingly abundant. Densities of >50 crabs per 100 m^2^ are not uncommon. The crabs (Fig. 1D) are omnivorous scavengers and feed upon fallen leaves, fruits, seeds, various types of plant debris, tree seedlings, and dead animals. Their presence on the forest floor has a considerable ecological impact by greatly reducing the extent of the litter layer and limiting the establishment of tree seedlings and other plants.

## RESULTS

**FIG 2:**
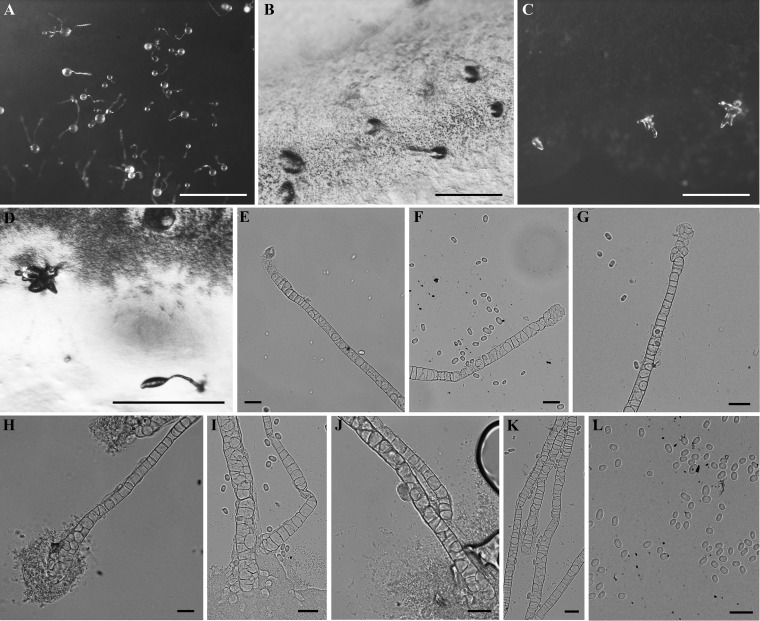
Morphological features of *Dictyostelium insulinativitatis*. (A) Sorocarps. (B) Aggregations. (C and D) Pseudoplasmodia and clustered sorogens. (E to G) Sorophore tips. (H to J) Sorophore bases. (K) Central parts of sorophores. (L) Spores. Bars: A, 1 mm; B to D, 500 µm; E to L, 20 µm.

**FIG 3:**
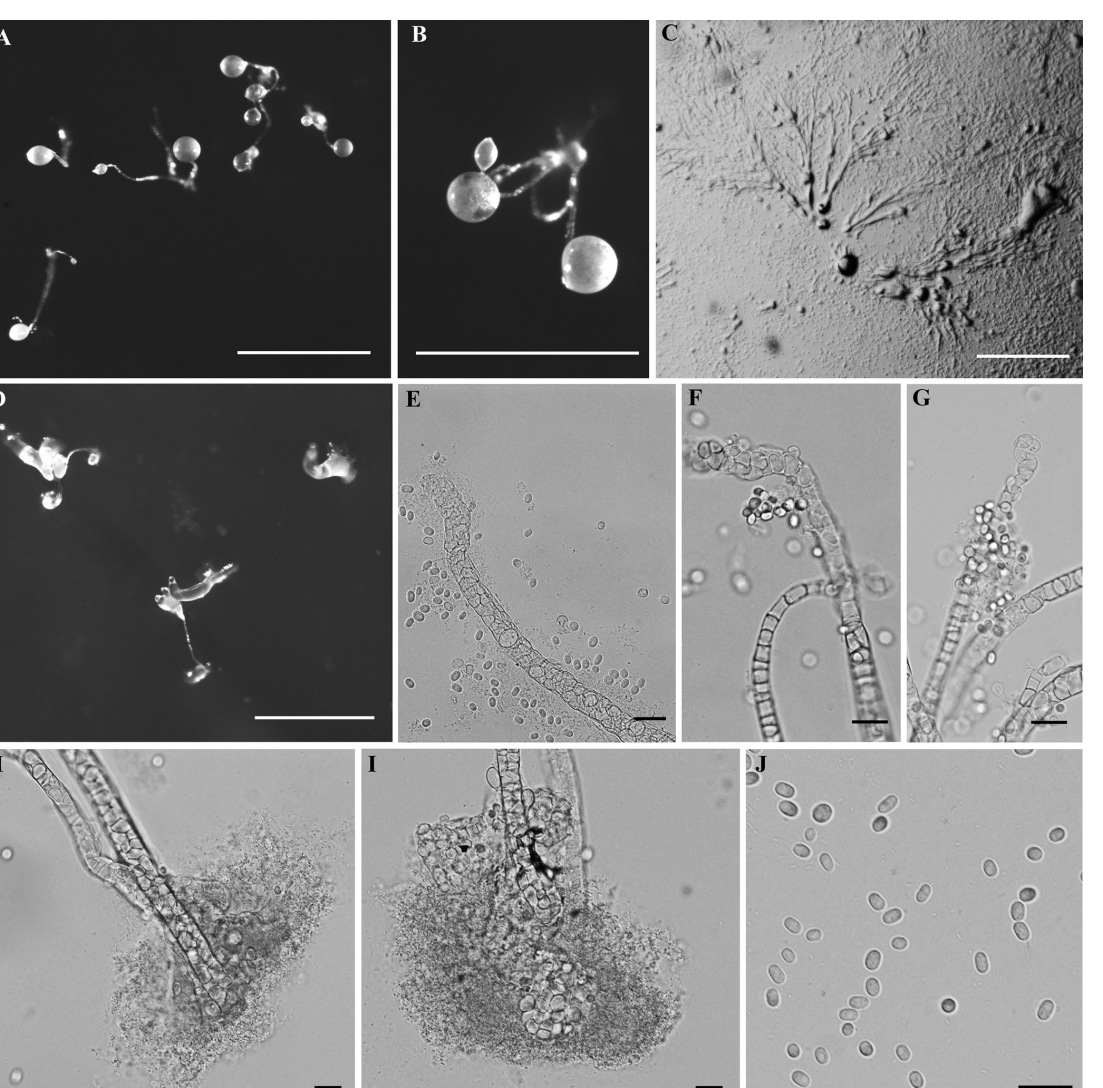
Morphological features of *Dictyostelium barbarae*. (A and B) Sorocarps. (C) Aggregations. (D) Pseudoplasmodia. (E to G) Sorophore tips. (H and I) Sorophore bases. (J) Spores. Bars: A to D, 1 mm; E to J, 20 µm.

**FIG 4:**
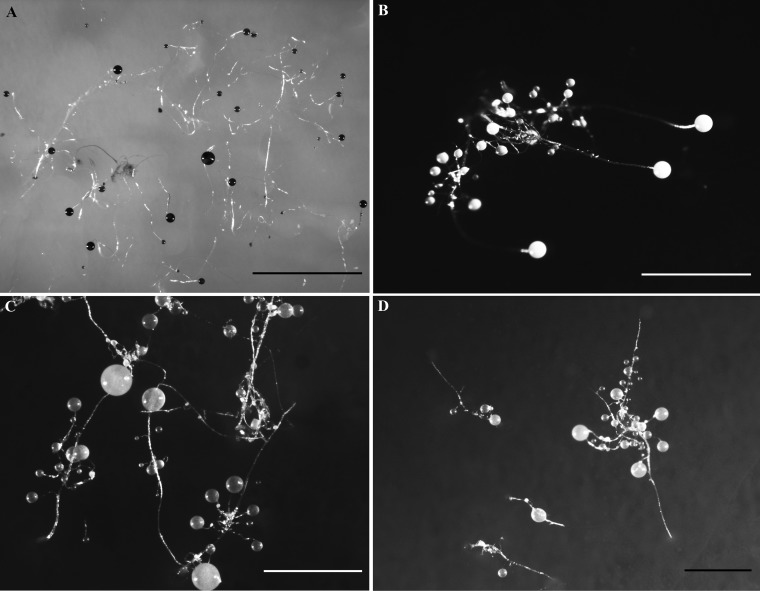
Other sorocarps. (A) *Dictyostelium purpureum* (isolate 2-1). (B to D) *Cavenderia aureostipes* [isolates 2-2, 2-3(1), and 2-3(2), respectively]. Bars: A, 5 mm; B to D, 1 mm.

**FIG 5:**
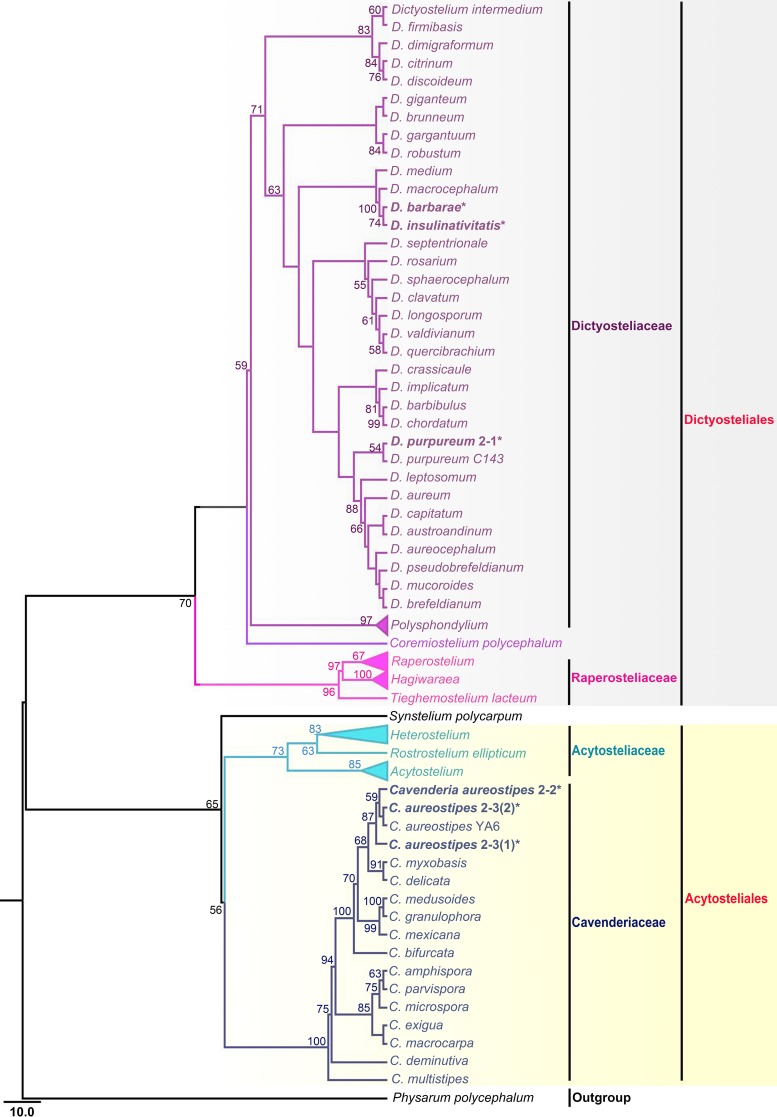
Phylogeny of four species obtained in this study along with other species of dictyostelids based on SSU rRNA, indicating the phylogenetic position of the new species *Dictyostelium insulinativitatis* and *D. barbarae*. Names with an asterisk are the sequences obtained in this study.

Six isolates representing four species of dictyostelids were recovered from samples collected at four localities on Christmas Island (Fig. 1A). Two isolates (Dictyostelium insulinativitatis and Dictyostelium barbarae) (Fig. 2 and 3) from one of the localities (L1) are new to science. Four isolates from a second locality (L2) were Dictyostelium purpureum Olive (isolate 2-1) and Cavenderia aureostipes (Cavender) S. Baldauf, S. Sheikh & Thulin [isolates 2-2, 2-3(1), and 2-3(2)] (Fig. 4). Two other localities (L3 and L4) did not yield any isolates. Phylogenetic studies of the ribosomal small subunit (SSU) further support the taxonomic placement of all four of the species recorded from Christmas Island (Fig. 5). Isolates of all of these species were deposited in the mycological herbarium of Jilin Agricultural University.

### Taxonomy and molecular phylogeny.

*Dictyostelium insulinativitatis* S. L. Stephenson, P. Liu, Y. Li et Y. Zou, sp. nov. (Fig. 2). MycoBank accession number MB829168. GenBank accession number MK322958. When cultured at 23°C on nonnutrient agar with Escherichia coli, sorocarps white, usually erect, gregarious, unbranched, commonly 0.45 to 1.26 mm high. Sorophore white, tips obtuse with one or two tiers of cells (6.99 to 9.03 µm in diameter), bases clavate with one or two tiers of cells (10.89 to 24.70 µm in diameter). Sori white, globose, commonly 0.11 to 0.15 mm in diameter. Spores hyaline, oblong to oval, 6.23 to 10.74 by 4.70 to 6.71 µm or 5.96 to 8.31 by 4.73 to 6.80 µm, without obvious polar granules. Aggregations mound-like, without radiate streams. Pseudoplasmodia migrate with stalk formation, producing clustered sorogens.

**(i) Etymology.** Referring to the locality (Christmas Island) where this species was isolated.

**(ii) Holotype.** HMJAU MR300 (strain 1-2) isolated from a soil/humus sample collected in an evergreen tall forest (L1, 10°28′24″S, 105°35′28″E), West White Beach Walking Track, off North West Point Road, 17 May 2017, Christmas Island, Indian Ocean.

**(iii) Comments.** This species belongs to dictyostelid group 4 (5) in an SSU ribosomal DNA (rDNA) phylogeny (Fig. 5). It forms a clade together with *D. barbarae* and Dictyostelium macrocephalum H. Hagiw. (6, 7). However, it differs morphologically from *D. barbarae* in the width of the sorophore. The sorophore of *D. barbarae* is composed of several tiers of cells, whereas that of *D. insulinativitatis* is made up of only one or two tiers of cells. The sorophore tip of *D. barbarae* is clavate or obtuse and wider than in *D. insulinativitatis*. Moreover, the base of the sorophore for *D. barbarae* is composed of several tiers of cells as opposed to only one or two tiers of cells in *D. insulinativitatis*. The spore size for *D. insulinativitatis* is larger than that of *D. barbarae*. The ratio of spore length and width in *D. barbarae* (1.5 to 1.63 or 1.05 to 1.38) is larger than in *D. insulinativitatis* (1.32 to 1.59 or 0.96 to 1.26). The habits of the sorocarps are clustered in *D. barbarae* and gregarious in *D. insulinativitatis*. The aggregation type of *D. insulinativitatis* is mound-like, but the aggregation type of *D. barbarae* is radiate with streams.

In addition, the sorocarps of *D. macrocephalum* (0.25 to 2.25 [−9.0]) are larger than this new species. The base of the sorophore in *D. macrocephalum* is conical, whereas the base of the sorophore in *D. insulinativitatis* is clavate. The ratio of spore length and width in *D. macrocephalum* (1.57 to 1.98) is larger than in the latter species (1.32 to 1.59 or 0.96 to 1.26). The aggregation of *D. macrocephalum* is radiate with streams, but aggregation in *D. insulinativitatis* is the mound-like type.

*Dictyostelium barbarae* S. L. Stephenson, P. Liu, Y. Li et Y. Zou, sp. nov. (Fig. 3). MycoBank accession number MB829169. GenBank accession number MK322959.

When cultured at 23°C on nonnutrient agar with E. coli, sorocarps white, usually erect, clustered, unbranched, commonly 0.22 to 1.59 mm high. Sorophore white with several tiers of cells, tips clavate or obtuse with one or two tiers of cells (9.87 to 14.83 µm in diameter), bases clavate or round with several tiers of cells (13.98 to 31.29 µm in diameter). Sori white, globose, commonly 0.120 to 0.202 mm in diameter. Spores hyaline, oblong or oval, 5.83 to 8.34 by 3.90 to 5.116 µm or 4.50 to 5.46 by 3.99 to 5.25 µm, without obvious polar granules. Aggregations radiate with streams. Pseudoplasmodia migrate with stalk formation.

**(i) Etymology.** Named in honor of the wife (Barbara Stephenson) of one of the coauthors of this paper.

**(ii) Holotype.** HMJAU MR301 (strain 1-5), isolated from a soil/humus sample collected in an evergreen tall forest (L1, 10°28′24″S, 105°35′28″E), West White Beach Walking Track, off North West Point Road, 17 May 2017, Christmas Island, Indian Ocean.

**(iii) Comments.** Molecular data from the ribosomal small subunit (SSU) support the placement of this species in the genus *Dictyostelium* (5) (Fig. 5). This species forms a clade with *D. insulinativitatis* and *D. macrocephalum*. The differences between *D. barbarae* and *D. insulinativitatis* were discussed in the comments provided for *D. insulinativitatis*. The sorocarps of *D. macrocephalum* (0.25 to 2.25 [−9.0]) are larger than in *D. barbarae*. The base of the sorophore in *D. barbarae* is clavate or round, whereas it is conical in *D. macrocephalum*. The ratio of spore length and width in *D. macrocephalum* (1.57 to 1.98) is larger than that in *D. barbarae* (1.5 to 1.63 or 1.05 to 1.38).

## DISCUSSION

Although long-distance dispersal by wind undoubtedly accounts for the presence of many of the microorganisms found on an isolated land mass such as Christmas Island, this is not necessarily true for all groups. For example, the spores produced by dictyostelids are embedded in a mucilaginous matrix that dries and hardens. As such, the spores stand little chance of being dispersed by wind (3, 8). However, as noted in the introductory section of this paper, numerous exotic plants have been introduced to Christmas Island, and it is certainly possible that some of these could have served as vectors for dictyostelids. Two of the four species (*Dictyostelium purpureum* and *Cavenderia aureostipes*) recovered in the present study are widely distributed throughout the world. The presence of the two species new to science is more problematic. Their occurrence may or may not be restricted to Christmas Island, since it is possible they can be found elsewhere but have not yet been reported. Conversely, these two species could be restricted to the island and thus provide evidence of the speciation process in dictyostelids. At this point, there is no way of knowing just which of the two situations exists.

In general, the overall diversity and abundance of dictyostelids on Christmas Island appear to be low. As noted above, two (L3 and L4) of the four localities yielded no isolates, and a total of only six clones were recovered from all 20 samples. For mainland Australia, the closest landmass for which comparable data exist, extensive sampling over a number of years (>300 samples from localities throughout the country) yielded an average of approximately 2.5 clones per sample (J. C. Landolt, unpublished data). The land crabs which are rather abundant in the forests on Christmas Island have a considerable ecological impact by reducing the extent of the litter layer. In some areas, very little litter exists. Since the soil-litter interface zone in forests represents the primary microhabitat for dictyostelids, any reduction in the amount of litter present on the forest floor might be expected to reduce the extent of this microhabitat and by extension the number of dictyostelids present. Interestingly, the most productive set of samples was obtained from the second locality (L2), a forest dominated by coconut palms. As noted earlier, coconut palm is an exotic plant that was introduced to Christmas Island.

For those species of dictyostelids present on Christmas Island, it seems likely that the land crabs have the potential of serving as vectors of their spores, since there is little question that as they move about, these animals invariably come into direct contact with the soil/litter microhabitat on the forest floor. The role of invertebrates in the dispersal of spores for dictyostelids is well established (9, 10).

There are few data relating to dictyostelids on isolated islands, but the total number of species (four) recorded in the present study is higher than the totals (three and one, respectively) reported for Ascension Island in the mid-Atlantic Ocean and Macquarie Island in the Subantarctic. However, these differences are not appreciable enough to be significant. Clearly, the dictyostelids of other small isolated oceanic islands need to be investigated in more detail.

## MATERIALS AND METHODS

### Sampling, isolation, and cultivation.

Twenty samples of soil/humus were collected from four localities on Christmas Island (Fig. 1A) in May 2017. Two of the localities (L1 and L4) were located in evergreen tall forests, one (L3) in a semideciduous closed forest, and one (L2) in a forest dominated by coconut palms. Each sample consisted of 10 to 20 g of soil/humus and was placed in a sterile Whirl-Pak plastic bag. The isolation methods used in the present study followed those described by Cavender and Raper (11) and are briefly summarized here. Each sample was weighed, and enough ddH_2_O water was added for an initial dilution of 1:10. This dilution was shaken to disperse the material and to suspend the amoebae, microcysts, and spores of dictyostelids. Afterward, an 0.5-ml aliquot of this dilution was added to each of five duplicate culture plates prepared with hay infusion agar (1). Approximately 0.4 ml of a heavy suspension of the bacterium E. coli was added to each culture plate as a food source. The plates were incubated at temperatures of 17 and 23°C with a 12-h light and dark cycle. Each plate was examined at least once per day for 2 weeks after the appearance of initial aggregations. Each isolate recovered from one of the plates was purified and cultivated for taxonomic studies and preservation on nonnutrient water agar plates with E. coli pregrown for 12 to 24 h. Spores from these plates were frozen in HL 5 medium (12) and stored at −80°C in the herbarium of the Mycological Institute of Jilin Agricultural University (HMJAU), Changchun, China.

### Observations of morphological features.

Six isolates were identified with the use of the descriptions provided by Raper (1) and molecular characteristics proposed by Sheikh et al. (5). First, the location of each early aggregating clone and sorocarp that developed in a plate was marked. The characteristic stages in the life cycle, including cell aggregation and the formation of pseudoplasmodia and sorocarps, were observed under a Zeiss dissecting microscope (Axio Zoom V16) with a 1.5× objective and a 10× ocular. Slides with sorocarps were prepared with water as the mounting medium. Features of spores, sorophores, and sorocarps were observed and measured on the slides by using a Zeiss light microscope (Axio Imager A2), with 10× ocular and 10, 40, and 100× (oil) objectives. Photographs were taken with a Zeiss AxioCam 506 color microscope camera.

### DNA isolation, PCR amplification, and sequencing.

The spores of all six isolates being studied were collected with a sterile tip and mixed with the lysis buffer of the MiniBEST universal genomic DNA extraction kit ver.5.0 (TaKaRa, Japan) according to the manufacturer’s protocol. The genomic DNA solution was used directly for the SSU PCR amplification using the primers 18SF-A (AACCTGGTTGATCCTGCCAG) and 18SR-B (TGATCCTTCTGCAGGTTCAC) (13) along with D542F (ACAATTGGAGGGCAAGTCTG) and D1340R (TCGAGGTCTCGTCCGTTATC) (14). PCR products were sent to Sangon Biotech Co., Ltd. (Shanghai, China), for sequencing. Sequences obtained were deposited in the GenBank database.

### Phylogenetic analysis.

The six newly generated sequences were checked and then submitted to GenBank. The SSU sequences were aligned and compared using the program MUSCLE v.3.6 (15, 16) and then manually adjusted in MEGA 7.0 (17). Maximum likelihood (ML) analyses were performed using RAxML v7 (18). In the ML analyses, the best-fit substitution models were estimated using the GTR submission model and a gamma correction for rate variation among sites (GTRGAMMA), using the CIPRES server. The statistical support of clades was assessed with 1,000 rapid-bootstrap (BS) replications.

### Nomenclature.

According to the International Code of Nomenclature used for algae, fungi, and plants, the electronic version of this article in portable document format (PDF) will represent a published work. In addition, new names contained in this study were submitted to MycoBank and allocated a unique MycoBank number which is accessible through MycoBank, Index Fungorum, GBIF, and other international biodiversity initiatives, where they are available to the Global Names Index.

### Data availability.

The isolates and the NCBI GenBank accession numbers of SSU DNA sequences considered in the present study are listed in Table 1. Sequence data are available in GenBank (accession numbers MK322958, MK322959, MK322960, MK322961, MK322962, and MK322963). The nomenclature of the new species in the present study is available in MycoBank (MB829168 and MB829169).

**TABLE 1:** NCBI GenBank accession information for SSU sequences of all isolates included in the phylogenetic analysis^*a*^

Taxon	Isolate no.	Accession no.
Acytostelium amazonicum	Landolt X	HQ141510.1
Acytostelium anastomosans	PP1	AM168115.1
Acytostelium digitatum	OH517	AM168114.1
Acytostelium leptosomum	FG12	AM168111.1
Acytostelium magnisorum	08A	HQ141513.1
Acytostelium serpentarium	SAB3A	AM168113.1
Acytostelium singulare	FDIB	HQ141514.1
Acytostelium subglobosum	LB1	AM168110.1
Cavenderia amphispora	BM9A	HQ141521.1
Cavenderia aureostipes	YA6	AM168083.1
**Cavenderia aureostipes**	**2-2**	**MK322961**
***Cavenderia aureostipes***	**2-3(1)**	**MK322962**
***Cavenderia aureostipes***	**2-3(2)**	**MK322963**
Cavenderia bifurcata	UK5	AM168084.1
Cavenderia delicata	TNS-C-226	AM168093.1
Cavenderia deminutiva	MexM19A	AM168092.1
Cavenderia exigua	TNS-C-199	AM168085.1
Cavenderia granulophora	CHII-4	AM168072.1
Cavenderia macrocarpa	MGE2	HQ141519.1
Cavenderia medusoides	OH592	AM168088.1
Cavenderia mexicana	MexTF4B1	AM168089.1
Cavenderia microspora	TNS-C-38	AM168090.1
Cavenderia multistipes	UK26b	AM168070.1
Cavenderia myxobasis	NT2A	HQ141522.1
Cavenderia parvispora	OS126	AM168091.1
Coremiostelium polycephalum	MY1-1	AM168056.1
Dictyostelium austroandinum		GQ496158.1
Dictyostelium aureocephalum	TNS-C-180	AM167876.1
Dictyostelium aureum	SL1	AM168028.1
***Dictyostelium barbarae***	**1-5**	**MK322959**
Dictyostelium barbibulus	Sweden-4R	JX173878.1
Dictyostelium brefeldianum	TNS-C-115	AM168030.1
Dictyostelium brunneum	WS700	AM168031.1
Dictyostelium capitatum	91HO-50	AM168032.1
Dictyostelium chordatum		GQ496159.1
Dictyostelium citrinum	OH494	AM168033.1
Dictyostelium clavatum	TNS-C-220	AM168035.1
Dictyostelium crassicaule	93HO-33	AM168037.1
Dictyostelium dimigraformum	AR5b	AM168038.1
Dictyostelium discoideum	V34	AM168039.1
Dictyostelium firmibasis	TNS-C-14	AM168041.1
Dictyostelium gargantuum		GQ496161.1
Dictyostelium giganteum	WS589	AM168042.1
Dictyostelium implicatum	93HO-1	AM168043.1
***Dictyostelium insulinativitatis***	**1-2**	**MK322958**
Dictyostelium intermedium	PJ11	AM168044.1
Dictyostelium leptosomum	NZN49A	HQ141480.1
Dictyostelium longosporum	TNS-C-109	AM168048.1
Dictyostelium macrocephalum	B33	AM168049.1
Dictyostelium medium	TNS-C-205	AM168050.1
Dictyostelium mucoroides	Sweden 20	HQ141482.1
Dictyostelium purpureum	C143	AM168060.1
***Dictyostelium purpureum***	**2-1**	**MK322960**
Dictyostelium pseudobrefeldianum	91HO8	AM168059.1
Dictyostelium quercibrachium	NZ201B	HQ141479.1
Dictyostelium robustum	TNS-C-219	AM168064.1
Dictyostelium rosarium	M45	AM168065.1
Dictyostelium septentrionale	IY49	AM168066.1
Dictyostelium sphaerocephalum	GR11	AM168068.1
Dictyostelium valdivianum		GQ496155.1
Hagiwaraea coeruleostipes	CRLC53B	AM168036.1
Hagiwaraea lavandula	B15	AM168047.1
Hagiwaraea radiculata	ML5A	HQ141494.1
Hagiwaraea rhizopodium	AusKY-4	AM168063.1
Hagiwaraea vinaceofusca	CC4	AM168062.1
Heterostelium anisocaule	NZ47B	AM168096.1
Heterostelium asymetricum	OH567	AM168097.1
Heterostelium australicum	NB1AP	HQ141508.1
Heterostelium candidum		AY040337.1
Heterostelium colligatum	HN13C1	HQ141505.1
Heterostelium equisetoides	B7JB	AM168099.1
Heterostelium filamentosum	SU-1	AM168100.1
Heterostelium flexuosum	AU4B	HQ141500.1
Heterostelium gloeosporum	TCK52	AM168074.1
Heterostelium luridum	LR-2	AM168101.1
Heterostelium multicystogenum	AS2	HQ141506.1
Heterostelium oculare		HQ141497.1
Heterostelium pallidum	TNS-C-98	AM168103.1
Heterostelium pseudocandidum	TNS-C-91	AM168107.1
Heterostelium rotatum	QC2C	HQ141501.1
Heterostelium stolonicoideum	K12A	HQ141507.1
Heterostelium tenuissimum	TNS-C-97	AM168105.1
Heterostelium tikalense	HN1C1	HQ141509.1
Heterostelium tikalense	OH595	AM168106.1
Physarum polycephalum	CL	X13160.1
Polysphondylium fuscans	Sweden-11D	JX173877.1
Polysphondylium laterosorum	AE4	AM168046.1
Polysphondylium patagonicum		GQ496156.1
Polysphondylium violaceum	209	HQ141486.1
Raperostelium australe	NZ80B	AM168029.1
Raperostelium gracile	TNS-C-183	AM168078.1
Raperostelium ibericum	214rjb	HQ141495.1
Raperostelium minutum	71-2	AM168051.1
Raperostelium monochasioides	HAG653	AM168052.1
Raperostelium ohioense	Okla4C	HQ141493.1
Raperostelium potamoides	FP1A	AM168069.1
Raperostelium tenue	PR4	AM168075.1
Rostrostelium ellipticum	AE2	AM168112.1
Synstelium polycarpum	VE1b	AM168057.1
Tieghemostelium lacteum		AM168045.1

a New sequences are indicated in bold.
